# Supplementary material for: Reconciling Mining with the Conservation of Cave Biodiversity: A Quantitative Baseline to Help Establish Conservation Priorities
Source: PLoS One. 2016 Dec 20;11(12):e0168348. doi: 10.1371/journal.pone.0168348 (PMC5173368; doi:10.1371/journal.pone.0168348)
Supplement: S1 Dataset — (ZIP) [file pone.0168348.s002.zip › Taxa/Serra Norte/SN_2013/N4WS/Lista N4WS-28.pdf]

CAVIDADE N4WS-0028

Classe	Ordem	Fam/Outros	Gên/Outros	Espécie	S-U	U-S	Zon.
Arachnida	Araneae	Ochyroceratidae		jovem	X		E
Arachnida	Araneae	Pholcidae	*Mesabolivar*	*eberhard*	X	X	E
Diplopoda	Polyxenida	Hypogexenidae		sp.1	X		E
Insecta	Diptera	Ceratopogonidae		jovem	X		E
Insecta	Heteroptera	Miridae		jovem	X		E
Insecta	Heteroptera	Reduviidae	Reduviinae	jovem		1	E
Insecta	Hymenoptera	Formicidae	*Brachymyrmex*	sp.1	X		E
Insecta	Hymenoptera	Formicidae	*Pheidole*	sp.4		X	E
Insecta	Lepidoptera	Tineoidea		sp.3		X	E
Insecta	Lepidoptera	Tineoidea		sp.6		X	E
Insecta	Neuroptera	Myrmeleontidae		jovem	1	2	E
Amphibia	Anura	Bufonidae	*Rhinella*	sp.		1	E
Mammalia	Chiroptera	Emballonuridae	*Peropteryx*	sp.	1		E
